# Paradoxical embolism after surgery for breast cancer: a case report

**DOI:** 10.1186/s12893-020-00798-5

**Published:** 2020-07-08

**Authors:** Zhimin Yu, Heran Deng, Jie Wang, Junyao Xu

**Affiliations:** 1 Department of hepatobiliary surgery, Sun Yat-Sen Memorial Hospital, Sun Yat-Sen University, #33 Ying feng Road, Guangzhou, P. R. China 510120; 2 Department of breast surgery, Sun Yat-Sen Memorial Hospital, Sun Yat-Sen University, Guangzhou, P. R. China 510120

**Keywords:** Paradoxical embolism, Pulmonary embolism, Renal artery embolism, Breast cancer, Surgery

## Abstract

**Background:**

Paradoxical embolism (PDE) presented with concomitant pulmonary embolism (PE) and renal artery embolism (RAE) which occurred to breast cancer patient after breast-conserving therapy, has never been reported.

**Case presentation:**

A 55-year-old female with breast cancer exhibited unexplained hypoxemia, followed with vomiting, diarrhea, unilateral flank pain and abdominal pain after lumpectomy 12 h. The urgent multi-detector row computed tomography (MDCT) confirmed the diagnosis of PE and RAE. Confusingly, the patient had no history of intracardiac defect, cardiac valvular diseases, atrial fibrillation or other cardiovascular disease and the definite cause was still unclear. However, after 10 days of prompt anticoagulant therapy in ICU, she was discharged in good condition.

**Conclusion:**

Breast cancer patients after surgery suffering from unexplained hypoxemia, abdominal pain, vomiting and diarrhea should be highly suspicious of PE or RAE, even PDE. Any clinical presentation on these postoperative patients should be given much more attention to make accurate diagnosis and appropriate interventions.

## Background

Paradoxical embolism (PDE) first proposed by Cohnheim in 1877, referring to the passage of venous or right-sided cardiac thrombus into the arterial or systemic circulation, is comparatively rare and represents less than 2% of all instances of systemic arterial emboli [1]. In general, the most common PDE sites are extremities (49%) and cerebrum (37%), where only 23% of PDE had two definable embolic sites and 10% had three [2]. Renal artery is infrequent and renal artery embolism (RAE) is typically seen on patient with atrial fibrillation or other cardiovascular disease [3]. It has been accepted that patent foramen ovale (PFO) or intracardiac defect working as a significant abnormal passage has played a crucial role in this process. However, in contrast to the accepted fact, the patient of this case without any indicator of most common risks still experienced concomitant PE and RAE after breast-conserving therapy.

## Case presentation

**Fig. 1:**
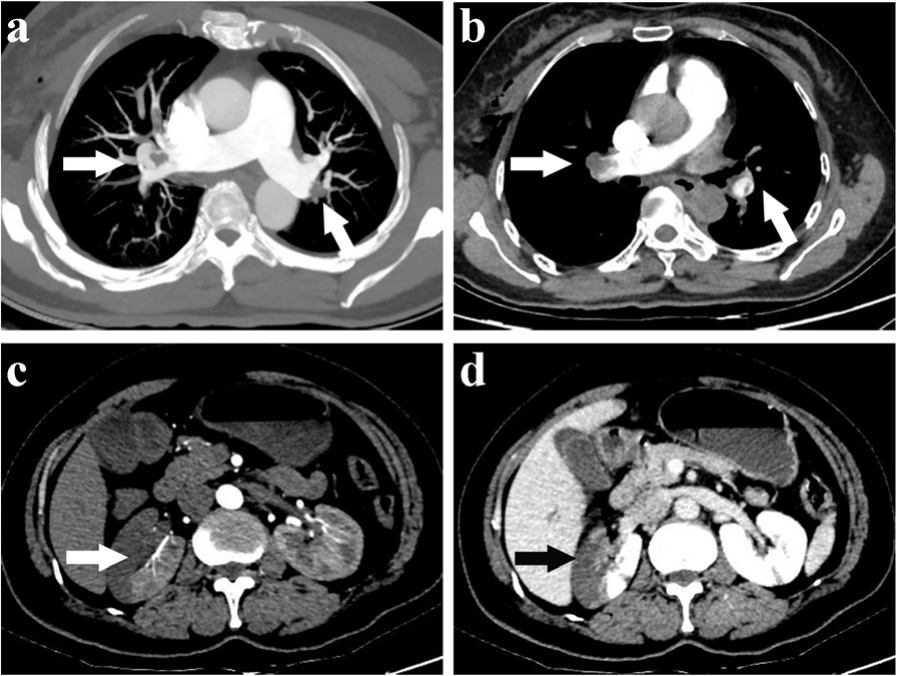
Contrast-enhanced CT angiography demonstrated a filling defect of main bilateral branches of pulmonary trunk (**a** and **b**), and an absent enhancement of a segment of right renal parenchyma in the upper pole (**c** and **d**). Ill-defined border and perinephric stranding suggested renal artery embolism was acute

**Fig. 2:**
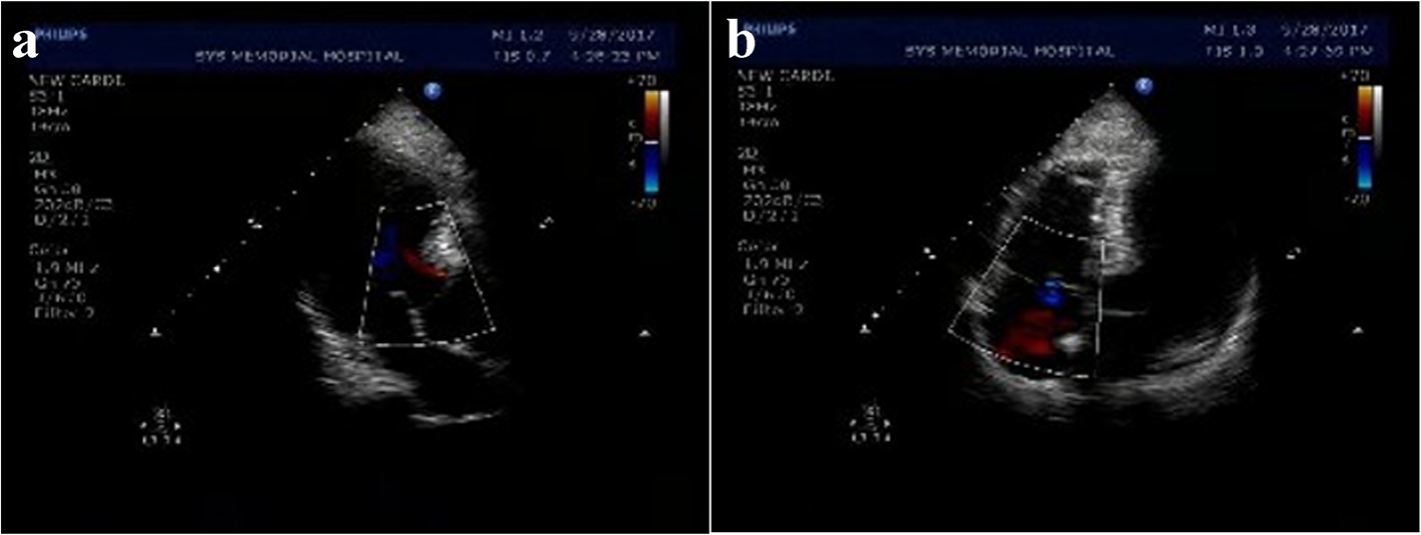
On the first day after paradoxical embolism event, the color doppler echocardiography revealed that there was no patent foramen ovale existence (**a**) or intracardiac defect except that the superior ventricular septum had a mild hypertrophy (15 mm) (**b**)

**Fig. 3:**
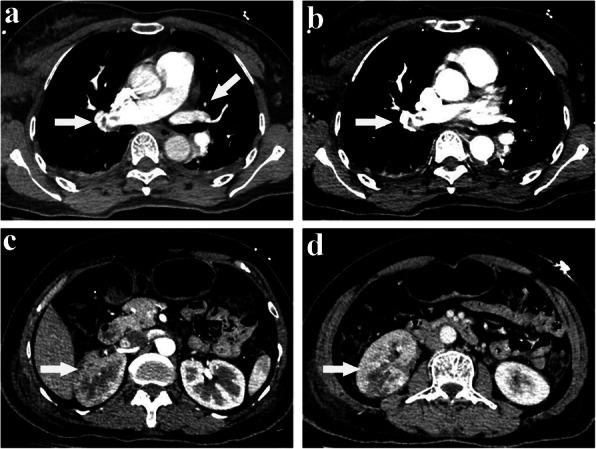
After seven days treatment of low molecular heparin anticoagulant therapy, Contrast-enhanced CT angiography revealed that most previous invisible thrombus in main bilateral branches of pulmonary trunk was dissolved and disappeared (**a** and **b**). Simultaneously, the blood flow perfusion of right kidney was restored without renal insufficiency (**c** and **d**)

A 55-year-old woman with BMI 24.6 kg/m^2^ diagnosed with invasive ductal carcinoma of right breast (cT2N0M0) was suddenly fainted with profuse sweating and followed developing severe gastrointestinal discomfort when she got up to walk in ward 12 h after lumpectomy, but no progressive chest pain, cough or unconsciousness was observed. Subsequently, she felt mild shortness of breath and dull pain on the right flank as well as lower right abdomen, accompanying vomiting and diarrhea in the next 30 min. Under this emergency circumstance, she was performed with a series of physical and screening laboratory examinations. Electrocardiogram monitor detected a mild decrease of blood oxygen saturation which ranged from 82 to 89% with 4 L of 100% oxygen inhalation through nasal cannula, blood pressure dropping to 88/57 mmHg, respiratory rate of 24/min, heart rate of 92 beats/minute with normal sinus rhythm. Apparent abdominal tenderness, rebound tenderness or abnormal auscultation findings was not detected on physical examination. The level of blood glucose was measured of 9.4 mmol/L. Brain natriuretic peptide did not indicate heart failure. Meanwhile, the value of myocardium enzymes including CK, CK-MB and cTnI were normal. D-dimmer level was slightly increased to 1.2 μg/ml (normal, < 1.0 μg/ml). The arterial blood gas analysis indicated: pH, 7.39; PaCO_2_, 42.9 mmHg; and PaO_2_, 65 mmHg. Additionally, routine urinalysis showed occult blood positive (+++) and microscopic haematuria was 120RBC/ul. Under 1000 ml liquid transfusion, there was still no any amelioration in her blood pressure and hypoxemia. After exclusion of hypoglycemia and acute myocardial infarction, the diagnosis of PE was highly suspected. Thromboembolism was evidenced in the main bilateral branches of pulmonary trunk and right renal artery (Fig. 1) after urgent MDCT of chest and abdomen. The final diagnosis was paradoxical embolism presented with concomitant pulmonary embolism (PE) and renal artery embolism (RAE). She was subsequently transferred to ICU and administered with anticoagulant therapy by low molecular weight heparin (LMWH, 4200 IU bolus) intravenously, followed by subcutaneously injection of LMWH (6000 IU) every 12 h upon the advice of multi-disciplinary team. Meanwhile, further examinations to unveil the cause of disease excluded the potential of antiphospholipid antibody syndrome (APAS), systemic vasculitis and other autoimmune diseases. Echocardiography showed no presence of PFO or intracardiac defect (Fig. 2), and no deep venous thrombus (DVT) of bilateral lower extremities was identified by ultrasound either. After 7 days of treatment with LMWH, most previous invisible thrombus could not be detected any more in the repeated MDCT scan (Fig. 3). Ultimately, she was discharged in good condition after 10 days therapy and advised to continuously take rivaroxaban for 3 months. During 3 months following up, she was doing well without any special complaint.

## Discussion and conclusion

Paradoxical embolism (PDE) is a potentially life-threatening complication in patients undergoing cancer surgery. It has been reported that the risk of PDE after DVT in patients with PFO is relatively lower which is less than 2% [4]. To the best of our knowledge, PDE presented with concomitant PE and RAE, which occurred to breast cancer patient after breast-conserving therapy, has never been reported. After all, the overall incidence of venous thromboembolism (VTE) was 0.16% after breast operation within 2 months [5].

**Fig. 4:**
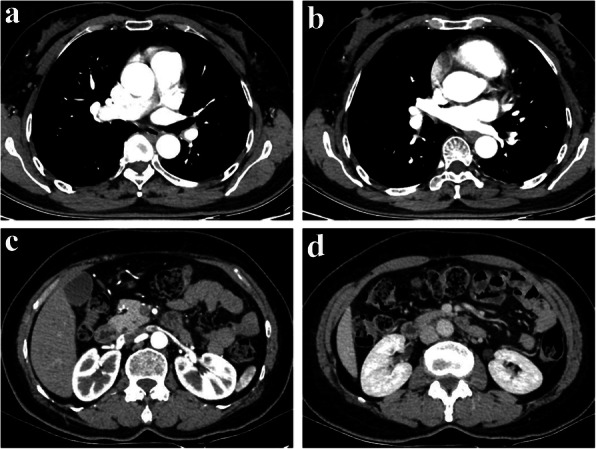
Preoperative contrast-enhanced MDCT of chest (**a** and **b**) and abdomen (**c** and **d**) indicated no thrombus or thrombosis was detected in pulmonary artery and renal artery of this patient

PDE usually occurs to patients with cardiovascular disease and VTE [2, 6]. Theoretically, a venous thrombus usually ends in the pulmonary artery or one of its branches, unable to cross the pulmonary capillaries and enter the systemic circulation unless an intracardiac communication and a favorable pressure gradient were presented [7]. The established evidence demonstrated that PFO has played a key role in PDE. Confusingly, in this case, the evidences of intracardiac defect, cardiac valvular diseases or abnormalities of visceral vessels in preoperative MDCT failed to be detected (Fig. 4). Hence, how DVT resulted in RAE was still unknown. The potential explanation was that PE resulted in transient pulmonary hypertension, which made invisible PFO reopened in a short time, eventually, incurred DVT into systemic circulation. If that is the case, why any abnormalities in echocardiograph or ultrasound was not detected? We speculated that the reason why any abnormalities in echocardiograph or ultrasound were not detected rested with the interval between the time of examinations and onset of the embolism event, which was similar to the experience of Travis JA [8]. Another additional reason was that we chose the echocardiograph to detect the existence of PFO other than transesophageal echocardiography mainly in consideration of severity of disease, which might affect the result to some extent.

For the breast cancer patients, there is no relationship identified between the stage of breast cancer or type of breast surgery and the development of VTE [5, 9]. However, this patient undergoing breast-conserving surgery without central venous catheter applied perioperatively and continuous compression of incision via elastic compression bandage postoperatively still had VTE. At present, the mechanisms of VTE responsible for cancer patient have not been clarified but thought to be correlated with hypercoagulable state of malignancy [10]. Three potential contributing factors should be given more focus and concern. The first one was associated with the nature of carcinoma. It’s estimated that cancer patients have a 4-fold increased risk of VTE comparing with general population, for an annual incidence of approximately 0.48% [11]. Moreover, a related study indicated that cancer patients have at least twice risk of developing postoperative DVT and over three times risk of fatal PE compared with non-cancer patients performed with the same surgical procedures [12]. Apart from that, the age and BMI(> 25 kg/m^2^), as two independent adverse prognostic factors of VTE, should also be taken account in this event [13].

Undoubtedly, compared with identifying VTE, identifying visceral artery embolism under initial stage is rather challenging. Similarly, RAE was difficult to be clinically diagnosed given its vague presentation [14]. In fact, it was gastrointestinal discomfort of this patient that caught our attention and raised clinical suspicion. Ultimately, we decisively adopted MDCT of abdomen to confirm the diagnosis of RAE. Thanks to the prompt diagnosis and effective therapy, she was free from distinct renal insufficiency with a value of serum creatinine and serum urea nitrogen fluctuation in normal range except the lactate dehydrogenase (range: 592-804 U/L; reference 108-252 U/L).

In conclusion, although the mechanisms responsible for increased risk of PDE in breast cancer patients are poorly understood, the unexplained hypoxemia followed by severe gastrointestinal discomfort, which exhibited postoperatively, should be highly considered as potential development of PE, even PDE. Keeping an open mind and attaching more attention to any clinical presentation of patients are indispensable to make an accurate diagnosis and give early appropriate interventions in clinical practice. Failing to do so may result in a considerable and incalculable adverse impact on patients. Moreover, defining patients at high risk will aid in establishing recommendations for PDE prophylaxis in the long run.

## Data Availability

All data analyzed was included in this published case report.

## Abbreviations

PE: Pulmonary embolism
RAE: Renal artery embolism
PDE: Paradoxical embolism
CTA: Computed tomography angiography
PFO: Patent foramen ovale
VTE: Venous thromboembolism
